# Ectopic tendons of the pectoralis minor muscle as cause for shoulder pain and motion inhibition—Explaining clinically important variabilities through phylogenesis

**DOI:** 10.1371/journal.pone.0218715

**Published:** 2019-06-21

**Authors:** Gilbert M. Schwarz, Lena Hirtler

**Affiliations:** 1 Department of Orthopedics and Trauma-Surgery, Medical University of Vienna, Vienna, Austria; 2 Division of Anatomy, Center for Anatomy and Cell Biology, Medical University of Vienna, Vienna Austria; University of Brescia, ITALY

## Abstract

**OJECTIVE:**

Clinical consequences of ectopic tendons of the pectoralis minor muscle (PMM) for shoulder pain and range-of-motion limitation have been demonstrated. For better understanding the existence of such ectopic tendons, a phylogenetic hypothesis is proposed.

**METHODS:**

Forty-five shoulders of anatomical specimens were dissected and examined. Insertions areas of PMM were measured and occurring aberrant tendons were identified. Their relationship with the coracohumeral ligament (CHL) described and samples of the ligament were collected and histologically stained.

**RESULTS:**

The prevalence of PMM variations was 37.84%. Shoulders with variations showed a statistically significant smaller coracopectoral distance (p<0.001) and larger insertion areas (p<0.003) than shoulders without. A strong negative correlation between these two variables (p<0.001, r = -0.620) was shown.

**CONCLUSIONS:**

The presented results prompted the conclusion that the CHL may be in fact the remnant of the pectoralis minor tendon (PMT), which migrated from the humerus to the coracoid process through the process of phylogenetic evolution. Variations of PMTs are significantly more common than in previous studies. Imaging techniques appear to be insufficiently sensitive for reliably detecting ectopic tendons. Especially in patients experiencing shoulder pain and stiffness in whom the commoner pathologies have been ruled out the possibility of ectopic PMT should be kept in mind and ruled out.

## Introduction

Aberrant tendons of the pectoralis minor muscle (PMM) have been demonstrated to play a role in the genesis of shoulder pain and range-of-motion (ROM) limitation. Involvement of this type of anatomical variations were noted in producing subacromial-, antero-medial subcoracoid impingement, shoulder stiffness, adhesive capsulitis, or superior labrum anterior to posterior (SLAP) lesions. [1–4]

**Fig 1:**
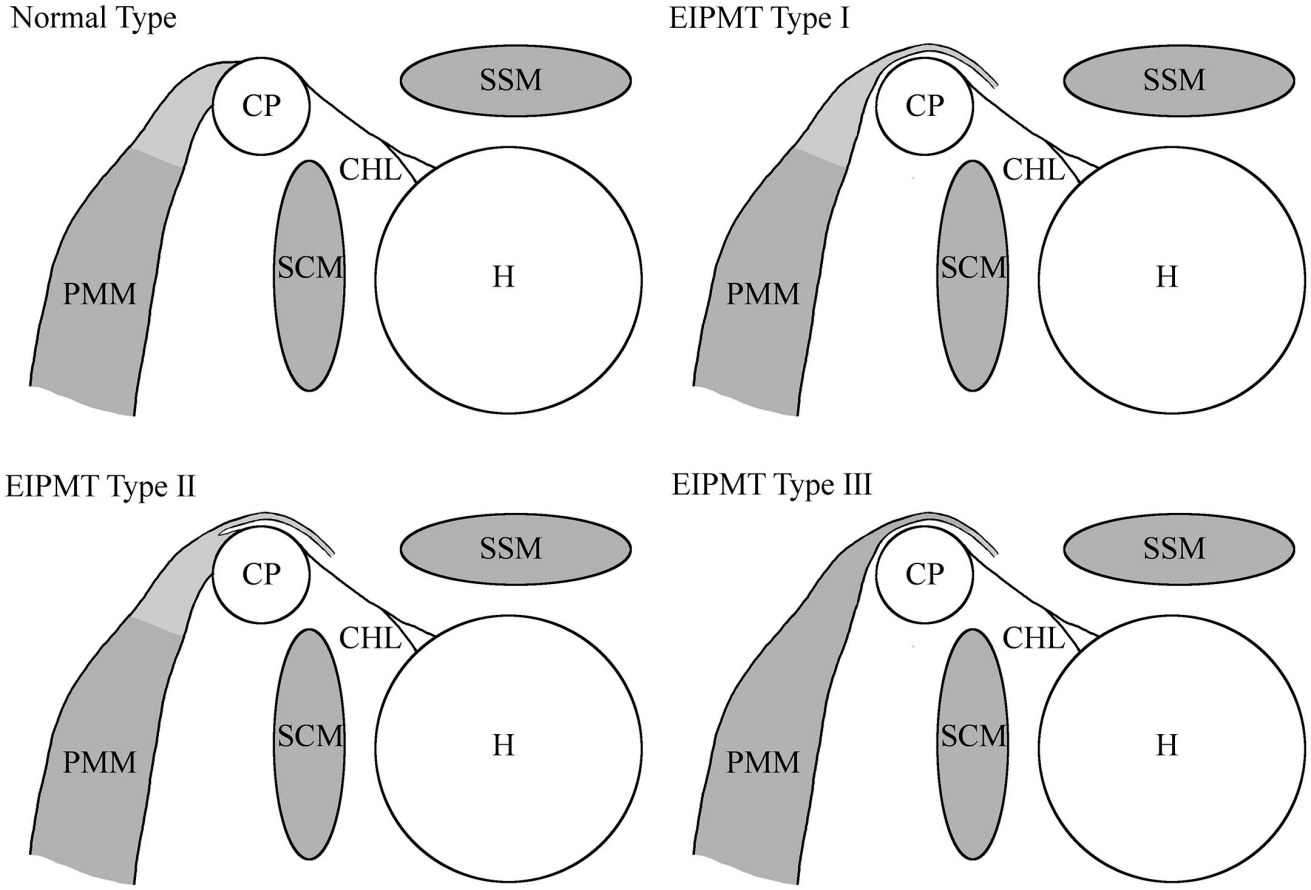
Schematic sagittal section of shoulders showing different kinds of EIPMTs according to Le Double. Modified from Lee 2014 [4]. CHL = coracohumeral ligament, CP = coracoid process, H = humerus, PMM = pectoralis minor muscle, SCM = subscapularis muscle, SSM = supraspinatus muscle.

Variations of the PMM were first described by Krause [5] and thereafter classified by Le Double [6]. Three different types of ectopic insertions of the pectoralis minor tendon (PMT) were delineated (Fig 1):

- Type I: The ectopic insertion of the pectoralis minor tendon (EIPMT) is located at the supraspinatus muscle, the greater or lesser tuberosity, the coracoacromial ligament (CAL), or the glenoid labrum and the tendon runs without attachment over the coracoid process. The passage between muscle and tendon is proximal to the coracoid process.
- Type II: One part of the PMT inserts at the coracoid process, and an aberrant part runs over the coracoid process and inserts at the greater tuberosity, the CAL or the joint capsule.
- Type III: The whole muscle (not merely the tendon) runs over the coracoid process and inserts at the greater or lesser tuberosity or the joint capsule.

Possible attachments of the ectopic tendons of the PMM included the joint capsule of the shoulder joint, the greater tuberosity of the humerus, the CAL, the tendon of the supraspinatus and coracobrachial muscles and the anatomical neck of the humerus. [1, 5, 7–12] Overall, such variability may among others be explained through embryological, generic or phylogenetic reasons. [13, 14]

Especially trying to explain the muscle’s variability of attachment through its phylogenetic origin, two main hypotheses may be mentioned: (1) The primary attachment of the PMM has been at the coracoid process. Through millennia and the evolution of man, the coracoid process became smaller in size, and the PMT therefore was required to find a new attachment location. Depending on its function in the corresponding species, its tendon migrated either to the humerus (carnivorous), the clavicle (rodent), or the scapula and fascia of the supraspinatus muscle (ungulate). [15] (2) The primary attachment of the PMM has been at the humerus. Due to its change of function over the course of evolution, its tendon migrated to the coracoid process, and the coracohumeral ligament (CHL) was determined to be its remnant. [16]

As general knowledge of clinically relevant anatomical variations is important, this study aimed to point out the relevance of functional and phylogenetic information on structures in the development of structural variability of the human body through the example of the attachment of the PMM. Additionally, such variations also influence the structure of neighboring structures, in this case the morphology of the CHL. The primary hypothesis was, that the morphology of the CHL changes if ectopic tendons of the PMM are present.

## Material and methods

A total of 53 formalin-phenol embalmed anatomical specimens (30 female, 23 male, age 83.21±10.06years) were examined. All specimens originated from voluntary body donations to the Center for Anatomy and Cell Biology of the Medical University of Vienna. All donors provided informed written consent prior to their death to have their bodies used in medical education and research. The study was approved by the Human Ethics Committee and the institutional review board of the Medical University of Vienna (No. 1501/2015).

Inclusion criteria were specimen availability and appropriate tissue quality. Exclusion criteria were severe degenerative changes to the shoulder joint and its surrounding structures or surgical procedures in the region. Eight specimens were excluded: complete rupture of the supraspinatus (n = 2) and PMT (n = 1), osteophytic lesions (n = 4), or total shoulder replacements (n = 1). Thus, 45 shoulders (10 paired, 33 unpaired -13 left, 20 right) remained to be examined.

**Fig 2:**
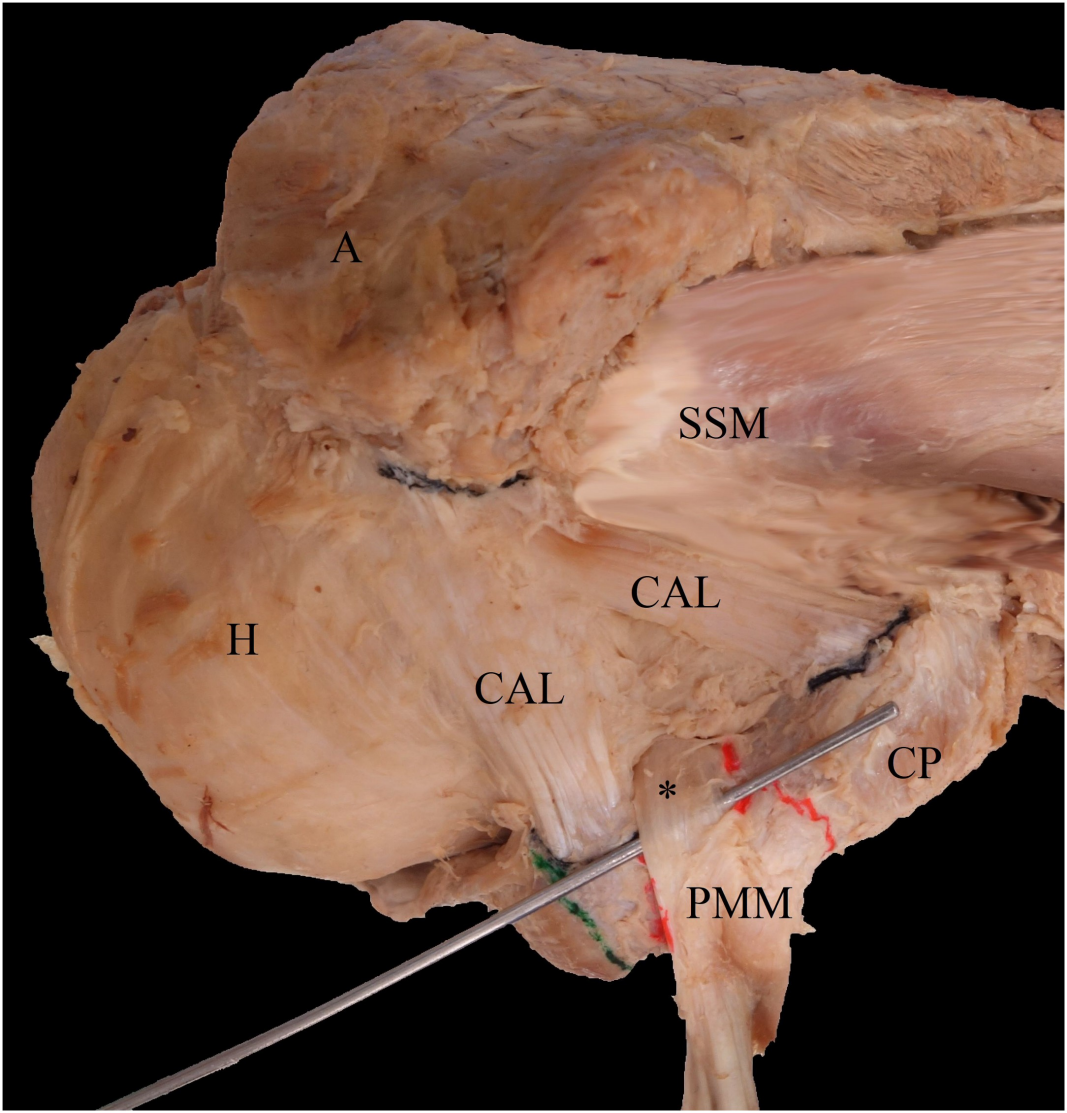
Exemplary photograph of a right shoulder seen from superior. The ectopic pectoralis minor tendon (*) runs over the coracoid process and through the branches of the coracoacromial ligament (CAL). A = acromion, CAL = coracoacromial ligament, CP = coracoid process, H = humerus, PMM = pectoralis minor muscle.

All specimens were mounted in a custom-made vice as commonly used in anatomical arthroscopic procedures. The coracoid process and its surrounding structures, including the PMM, the coracobrachial, and biceps brachii muscles, the CAL, the CHL, and the muscles of the rotator cuff were carefully dissected (Fig 2).

**Fig 3:**
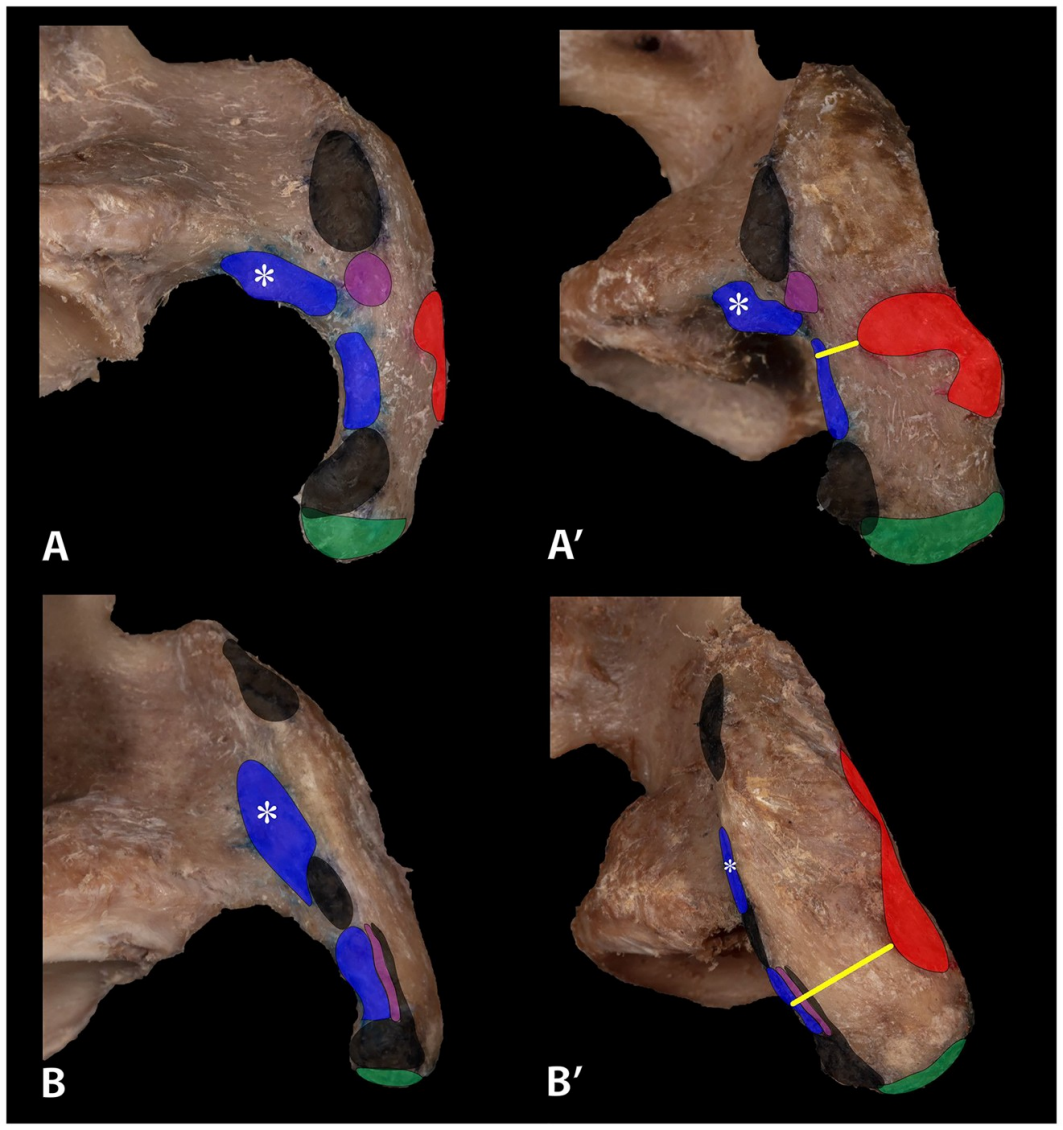
Right EIPMT (A and A’) and normal (B and B’) type shoulder from cranial (A and B) and lateral (A’ and B’). Insertion areas are marked on the coracoid process. Red = PMM, black = CAL, blue = CHL, * medial CHL fibers, violet = CGL, green = coracobrachialis muscle/short head of biceps. Insertion areas of the PMM in EIPMT type shoulders are significantly bigger (red) and coracopectoral distances (yellow line) to the lateral border significantly smaller. In EIPMT type shoulders with tendons blending into the CHL the CGL insertion area must move more proximal and the CAL must be divided into two branches to give space to the CHL.

Insertion areas of the PMM, CHL and coracoglenoidal ligament (CGL) on the coracoid process as well as length and width of the horizontal part of the process were marked, measured, and recorded in a standardized fashion. The distance from the insertion area of the PMM to the lateral border of the coracoid process was measured and defined as the coracopectoral distance (Fig 3, yellow line). The course of ectopic PMT and its relationship with the CHL were noted and its width was measured at the lateral border of the coracoid process.

Tissue samples from the CHL (5mm diameter) were taken 1 cm distal to the lateral border of the coracoid process and evaluated histologically (resorcin-fuchsin and picrosirius-red staining). The samples were embedded in paraffine and cut with a thickness of 10μm.

Statistical analysis was performed with IBM SPSS Statistics software (BM Corp. Released 2018. IBM SPSS Statistics for Windows, Version 25.0. Armonk, NY: IBM Corp.). Descriptive statistics (mean, SD, minimum and maximum) were computed for all metric variables. Differences in sex, arm dominance, insertion area size, and coracopectoral distance between shoulders with ectopic insertions of the PMM and normal type insertion groups were evaluated. For normally distributed metric data a Student’s t-test and for non-normally distributed variables a Mann-Whitney U-test was used. For categoric variables, qui-square test was performed. Spearman rank coefficient was used for normally distributed data (0.8<r≤1.0, strong; 0.5<r ≤0.8, moderate; 0.2< r ≤0.5, weak; 0.0< r ≤0.2, no correlation). To account for the different sizes of examined scapulae and the hypothesis of Lander[15], each insertion area (PMM, CHL) was put in relation to its coracoid process by dividing length and width of the coracoid process through PMM or CHL area size. A p-value <0.05 was considered as significant. Bonferroni correction was applied in multiple testing.

## Results

Table 1 shows dimensions and relationships between shoulders with (EIPMT type, n = 18, 40%, Fig 2) and without (Normal type, n = 27, 60%) ectopic tendons.

**Table 1:** Dimensions of and relationships between shoulders with (EIPMT) and without (Normal) ectopic insertions. CGL = coracoglenoid ligament CHL = coracohumeral ligament, PMM = pectoralis minor muscle.

	Total	EIPMT group	Normal group
**Number**	45	18 (40%)	27 (60%)
**Gender**			
Female	28 (62%)	13 (46%)	15 (54%)
Male	17 (38%)	5 (29%)	12 (71%)
**Side** ^1^			
Left	19 (42%)	11 (58%)	8 (42%)
Right	26 (58%)	7 (27%)	19 (73%)
**PMM insertion area (mm**^**2**^**)** ^2^	119.2±40.6	132.2±37.9	110.5±40.7
**Coracopectoral distance (mm)** ^2^	7.9±2.9	5.6±2.8	10.1±2.9
**Length of coracoid process (mm)**	47.1±4.4	45.7±3.9	48.0±4.4
**Width of coracoid process (mm)**	15.6±2.7	14.8±2.7	16.1±2.6
**Distance from tip of coracoid process to CGL (mm)** ^3^	11.6±5.3	14.5±5.5	9.4±5.1
**CHL insertion area (mm**^**2**^**)**	56.5±36.3	66.31±33.8	54.9±34.4

^1^ left arm dominance in EIPMT group statistically significant (p = 0.036)

^2^ significant difference between EIPMT group and normal group (p<0.001)

^3^ significant difference between EIPMT group and normal group (p = 0.036)

Results indicated a female and left arm predominance with left arm dominance being significant (p = 0.036) and female not (p = 0.259). All EIPMT shoulders had a primary insertion at the coracoid process and an accessory tendon running over the coracoid process at the CHL (n = 11), CGL (n = 11), glenoid labrum (n = 4), or at the capsule (n = 2), all classified as type II based on Le Double. [6] The mean width of the accessory ectopic tendon was 3.62±1.87mm. For tendons blending into the CHL the mean width was 4.5±1.8mm and for tendons running into the CGL 2.5±0.9mm (not significant = n.s.).

In all examined shoulders, the PMM inserted at the medial and, to a varying extent, the upper surface of the coracoid process. The discrepancy in PMM insertion size between EIPMT shoulders and normal type shoulders was significant (p = 0.001).

The coracopectoral distance differed significantly between normal and EIPMT type shoulders (p<0.001). Correlation analysis using Spearman’s rank-order between insertion areas of the PMM and the coracopectoral distance demonstrated a strong negative correlation (p<0.001, r = -0.620).

Examination of the lateral surface revealed a specific arrangement of inserting structures in all specimens. From cranial to caudal the CAL, CHL and CGL were detected in that specific order (Fig 3). While the CAL and CHL were present in all examined shoulders, the CGL could only be demonstrated in 71.43% (n = 32). EIPMT type shoulders with tendons blending into the CHL showed a more proximal placement of the CGL (Fig 3) relative to normal type shoulders (p = 0.036). When ectopic tendons blended with the CGL, the distance from the tip of the coracoid process was similar between normal type and EIPMT shoulders (p = 0.847).

**Fig 4:**
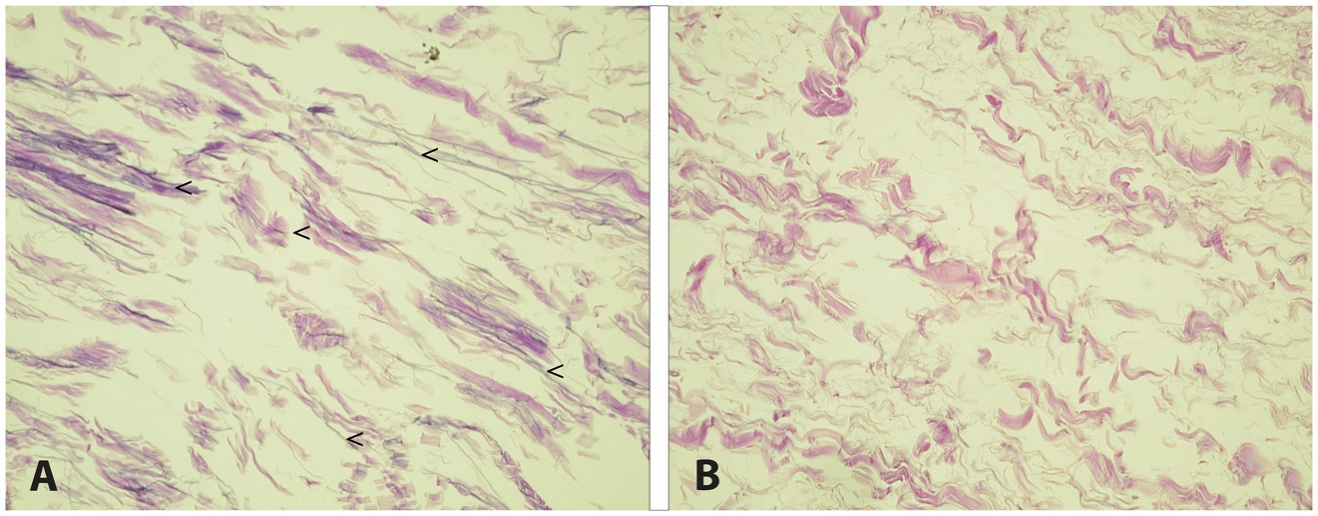
Coracohumeral ligament (CHL). CHL retrieved from shoulders with EIPMT (A) showed a higher number of specimens with large amounts of elastic fibers (arrowhead) compared to normal shoulders (B). (resorcin-fuchsin stain, original magnification x20).

**Fig 5:**
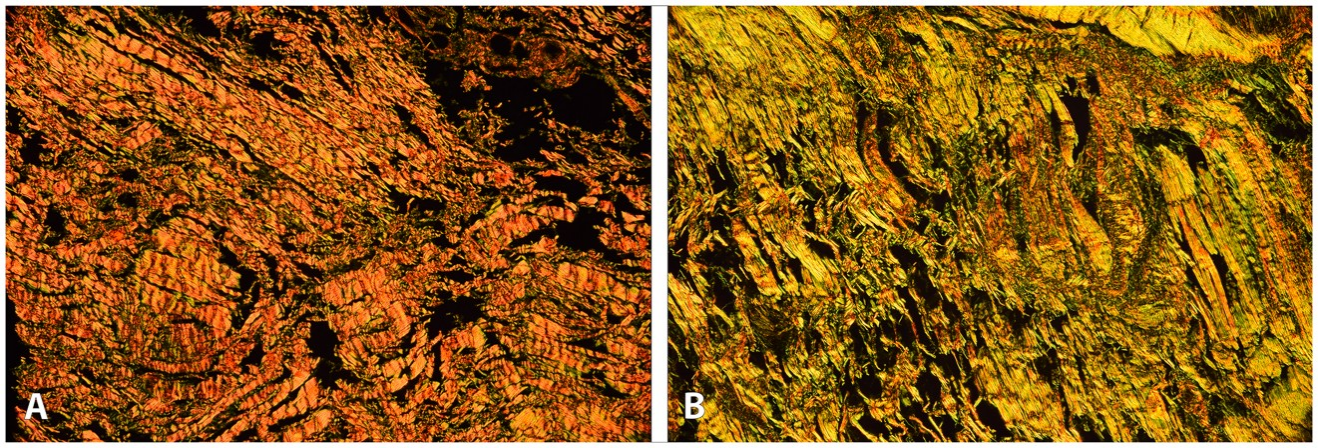
Coracohumeral ligament (CHL). CHL retrieved from shoulders with EIPMT (A) showed a higher number of specimens with predominant type I collagen fibers (orange) compared to normal shoulders (B, predominant collagen type III, yellow-green). (picrosirius-red, original magnification x10, polarized).

The CHL was present in all specimens and originated from the lateral aspect of the coracoid process. There was no significant difference between EIPMT and normal type shoulders. Staining with resorcin-fuchsin for displaying elastic fibers demonstrated a higher number of specimens with elastic fibers in the EIPMT group (85%) relative to normal type shoulders (35%) (Fig 4). EIPMT group shoulders displayed a higher number of specimens with more type I collagen fibers (67%), while normal type shoulders displayed a higher number of specimens with more type III collagen fibers (54%). This difference was evaluated through picrosirius-red staining in combination with polarized light. Then, thicker fibers (type I collagen) are characterized by a yellow-orange birefringence and thinner fibers (type III collagen) are characterized by a green birefringence (Fig 5).

In 46.7% (n = 21) additional fiber bundles medial to the CHL were present. (Fig 3) All of them were blending with fibers of the glenohumeral capsule and showed to have a high number of type I collagen fibers (80%) and elastic fibers (66.67%).

## Discussion

This study is one of the few in literature to evaluate the prevalence of ectopic PMM insertions and its phylogenetic entity in human anatomical specimens. It illustrates that ectopic tendons running over the coracoid process (Fig 2) influence the morphology of the CHL and are more frequent than recent literature has contended [3, 4, 17] (Table 2), which is of clinical importance evaluating differential diagnoses in unclear shoulder pain. In addition, while recent studies exclusively applied image techniques, such as US, MR, or MR arthrography, this study detected ectopic fibers through anatomical dissection, reflecting a more reliable cross-sectional information on the general population.

**Table 2:** Prevalence and Le Double classification relative to previous studies. N = number of included shoulders.

	Study design	N	Prevalence	Le Double
**Present study**	Prospective anatomical dissection	45	37.84%	Type II
**Fang-Dschau 1937** [8]	Prospective anatomical dissection	88	34%	Type I 33.3% Type II 66.6%
**Homsi 2003** [17]	Ultrasound study	606	9.57%	---
**Lee 2014** [4]	Retrospective MRI analysis	507	13.40%	Type I 7.7% Type II 5.7%
**Lee 2010** [3]	Retrospective MR-arthrography analysis	335	1.5%	Type III

### Prevalence of ectopic tendons of the PMM

Scarce information on the prevalence of anomalous insertions of the PMM may be found in literature (Table 2). The results were based on anatomical dissection [8] or imaging modalities as ultrasound [17], MR arthrography [3] and standard MRI [4, 18]. Ectopic tendons were found in 1.5–34% of the evaluated cases [3, 4, 8, 17, 18]. Information on their specific LeDouble classification [6] were only available in three of those papers, reporting LeDouble type I in 7.7–33.3% [4, 8], LeDouble type II in 5.7–66.6% [4, 8] and LeDouble Type III in 1.5% [3]. A left-side [3, 4, 17] and female predominance [17] was reported similarly to the results of the present study.

These large ranges of prevalence could be explained by the small size of ectopic tendons and their difficult detection in imaging modalities. In direct comparison between MRI and arthroscopy, the sensitivity and specificity of MRI were reported as 64% and 80%, respectively. In combination with a false positive value of 70% and a moderate interobserver reliability (0.437–0.532), this highlights the potential of misinterpretation in imaging modalities. [18]

In present study, all EIPMT-shoulders were classified as Le Double type II. As type III variations were previously only demonstrated in one study [3] and have not been demonstrated otherwise, a sole differentiation between type I and type II as previously proposed by Lee et al. [4] seems sufficient.

The CHL will be influenced in its morphology by ectopic tendons of the PMM, which may lead to the large variety of different attachments described in literature: greater and lesser tubercle, glenohumeral capsule, rotator interval or the tendons of the rotator cuff [19–23]. However, a precise distinction of the CHL is hardly ever possible [24], as the results confirmed. The CHL therefor should be defined as the complex of fibers located in the rotator interval with variable humeral insertions, compared to the textbook description of its V-shaped course, directing the long biceps tendon along the intertubercular groove, as this is the exception rather than the rule.

Also, the insertions of structures on the lateral facet of the coracoid process vary in EIPMT type shoulders (Fig 3). In cases with ectopic tendons blending into the CHL, the insertion area of the CGL moved more proximal. As ectopic tendons running into the CHL were bigger (4.5mm) than tendons blending into the CGL (2.5mm), this proximal placement could be explained by the larger space ectopic tendons to the CHL required.

### Phylogenetic origin of ectopic tendons of the PMM

Ectopic PMT were coursing through or medial to the fibers of the CAL and merged into the CHL or CGL. According to the three elementary rules about the nature and morphology of ligaments proposed by Bland Sutton [25], it is highly probable that the CHL is the remnant of the PMT: (1) The CHL corresponds with the PMT in origin and insertion; (2) The PMM shows anatomical variation in humans; (3) and the PMM in species with an insertion at the humerus assumes a functional importance.

Negative correlation between the size of PMT insertions areas and the coracopectoral distances have been demonstrated, suggesting a phylogenetic migration from the humerus to the coracoid process of the scapula, as theorized by Bland Sutton [25], Rouvière and Delmas [16], and Tubbs et al. [9]. Adding to this hypothesis of evolutional migration is the fact that despite the immediate proximity between the CHL and CAL no ectopic fibers could be found blending into the CAL.

Also, the microscopic evaluation of the CHL in EIPMT type shoulders showed a morphological difference on the microscopic level. They appeared to have more type I collagen and elastic fibers relative to normal shoulders.

### Clinical relevance

The association of EIPMT type shoulders and pain was already described in literature. In patients with therapy-resistant ROM limitation in combination with shoulder pain one of the differential diagnoses to consider are ectopic tendons of the PMM. [1–3, 10] They may impinge, have an impact on fibrotic scar tissue proliferation in the rotator interval and also lead to a higher prevalence of SLAP lesions. [2–4] The present study aimed to establish clarity regarding the real in vivo prevalence of EIPMT type shoulders with a profound anatomical description of size and course of ectopic PMT. It is in the nature of anatomical studies using post mortem retrieved specimens, that clinical conclusions must be drawn carefully. However, the presented results lead to the conclusion that ectopic PMTs may have clinical significance in patients experiencing shoulder pain and stiffness in whom the commoner pathologies have been ruled out. Patients with a SICK scapular syndrome (i.e. Scapular malposition, Inferior medial border prominence, Coracoid pain and malposition, and dysKinesis of scapular movement) often present with coracoid pain due a tightened PMM, which tilts the coracoid inferiorly. [26] Ectopic PMTs may change the shoulders kinetic chain and explain why not all patients complain of coracoid pain. As this study was also able to show, preoperative imaging such as MRI and ultrasound seem to be insufficiently sensitive to detect ectopic tendons of PMMs. Adding to that it seems unlikely that all ectopic PMT lead to the reported symptoms. With the utmost probability certain size and course (into CHL more likely than into CGL) of ectopic tendons are necessary to cause complaints with limited ROM and shoulder pain as already described in literature. [1]

### Limitations

The age of examined anatomical specimens and their formalin-phenol fixation could be named as limiting factors of this study. Most shoulders showed signs of degeneration of the joint and the surrounding tissue. Through careful definition of the exclusion criteria, dissection of the CHL at the coracoid process was nonetheless possible. Due to the alteration of formalin-phenol fixated specimens, dissection of distal coracohumeral fibers was aggravated, and no biomechanical or clinical conclusions can be drawn. Since ectopic tendons may cause decreases in ROM, [1] further anatomical and biomechanical studies of fresh-frozen anatomical specimens should be conducted to clarify what size of ectopic tendons would be clinically relevant. The high prevalence discovered in the present study supports the approach of underlining the importance of recognizing these common abnormal findings in patients with stiff shoulders and therapy-resistant shoulder pain.

### Conclusion

The presented results prompted the conclusion that the CHL may be in fact the remnant of the PMT, which migrated from the humerus to the coracoid process through the process of phylogenetic evolution. Variations of PMTs, with a prevalence of 37.84%, are significantly more common than in previous studies. Imaging techniques (US, MRI, MR-arthrography) appear to be insufficiently sensitive for reliably detecting EIPMT. Especially in patients experiencing shoulder pain and stiffness in whom the commoner pathologies have been ruled out the possibility of ectopic PMT should be kept in mind and ruled out.
